# Mechanisms of enhancer-promoter communication and chromosomal architecture in mammals and *Drosophila*

**DOI:** 10.3389/fgene.2022.1081088

**Published:** 2022-12-01

**Authors:** Olga V. Kyrchanova, Oleg V. Bylino, Pavel G. Georgiev

**Affiliations:** Department of the Control of Genetic Processes, Institute of Gene Biology Russian Academy of Sciences, Moscow, Russia

**Keywords:** TAD, distance interactions, architectural C2H2 proteins, zinc-finger proteins, homodimerization domains, cohesin, CTCF

## Abstract

The spatial organization of chromosomes is involved in regulating the majority of intranuclear processes in higher eukaryotes, including gene expression. Drosophila was used as a model to discover many transcription factors whose homologs play a key role in regulation of gene expression in mammals. According to modern views, a cohesin complex mostly determines the architecture of mammalian chromosomes by forming chromatin loops on anchors created by the CTCF DNA-binding architectural protein. The role of the cohesin complex in chromosome architecture is poorly understood in Drosophila, and CTCF is merely one of many Drosophila architectural proteins with a proven potential to organize specific long-range interactions between regulatory elements in the genome. The review compares the mechanisms responsible for long-range interactions and chromosome architecture between mammals and Drosophila.

## 1 Introduction

The sets of transcription factors (TFs) assembled on regulatory genome elements, enhancers and promoters, determine cell specialization in higher eukaryotes. Promoters are up to 150 bp in size and contain motifs for DNA-binding TFs that open chromatin and ensure recruitment of general TFs that determine start site and direction of transcription (Vo Ngoc et al., 2019; Andersson and Sandelin, 2020). Enhancers are approximately 500 bp on average and consist of combinations of motifs recognized by DNA-binding TFs (Cavalheiro et al., 2021; Lim and Levine, 2021; Serebreni and Stark, 2021). Dozens of enhancers often regulate transcription of developmental genes and some of them are hundreds of kilobases away from their target promoters (Furlong and Levine, 2018). Special regulatory elements known as insulators maintain the specificity of enhancer–promoter interactions (Melnikova et al., 2020). Insulators partly or completely block the interactions between a promoter and an enhancer when located between them in transgenic lines. In some cases, insulators were found to form chromatin loops to support long-range interactions between regulatory elements. The current review compares the mechanisms of long-range interactions between mammals and *Drosophila* based on the available models and experimental data.

## 2 General concepts of topologically associated domains and mechanisms of interaction between enhancers and promoters

Whole-genome Hi-C studies in various organisms and cell lines showed that chromosomes are divided into large domains wherein contacts between distant chromatin regions occur at similar frequencies (Dixon et al., 2012; Nora et al., 2012; Sexton et al., 2012; Ulianov et al., 2016). On average, half the frequency of contacts is observed for DNA regions located on opposite sides of the domain boundary. The domains were termed the topologically associated domains (TADs). Complex regulatory regions of developmental genes and genes similar in expression pattern occur mostly within TADs (Sikorska and Sexton, 2020; Jerkovic and Cavalli, 2021). TADs are now thought to provide a universal structural unit of chromosome organization not only in mammals, but, possibly as certain variants, in all eukaryotes. Patterns of TADs differ between Drosophila and mammals. In mammals, TAD boundaries are mostly outside genes and co-localize with sites for the CTCF architectural protein and the cohesin complex (Rao et al., 2014). A higher contact frequency of relatively close internal regions is observed at TAD boundaries, producing a dot on a 2D heat map. Dots were experimentally attributed to CTCF and the cohesin complex, which form the majority of mammalian TADs (Nora et al., 2017; Rao et al., 2017). However, recent single-cell Hi-C studies (Arrastia et al., 2022) and high-resolution microscopy (Szabo et al., 2020) have shown that in a large part of TADs, the borders do not have a clear localization. For an illustrative example, the Fbn2 gene TAD, which is one of the most stable TADs, was studied in mouse embryonic stem cells (ESCs) by super-resolution microscopy (Gabriele et al., 2022). A chromatin loop between the TAD boundaries with CTCF sites was found to form in 6.5% of nuclei and to live no more than 30 min on average. Thus, TAD boundaries form as a set of preferential interactions rather than as a stringent physical barrier that blocks any trans-interactions between regulatory elements (Chang et al., 2020; Luppino et al., 2020; Sikorska and Sexton, 2020; Szabo et al., 2020).

In *Drosophila*, TAD boundaries most often coincide with housekeeping gene clusters and lack sites to preferentially bind a single architectural protein, such as CTCF in mammals (Ramírez et al., 2018; Wang et al., 2018). A dot pattern is usually not produced on a 2D heat map by TAD boundaries, suggesting relatively rare interactions over extended boundary regions. This is consistent with a high heterogeneity of contacts observed at *Drosophila* TAD boundaries by single-cell Hi-C (Ulianov et al., 2021) and high-resolution microscopy (Szabo et al., 2020). The role of TAD chromosome organization in gene expression is unknown. Gene transcription within disrupted TADs remained virtually unchanged in *Drosophila* lines carrying multiple inversions and deletions (Ghavi-Helm et al., 2019).

Tethering elements (TEs) were identified as new regulatory elements in *Drosophila* by micro-C analysis, which detects long-range contacts in the genome to high resolution. Stable chromatin loops are formed by TEs (Batut et al., 2022; Levo et al., 2022). Only part of TEs coincide with TAD boundaries, enhancers, and promoters.

**FIGURE 1:**
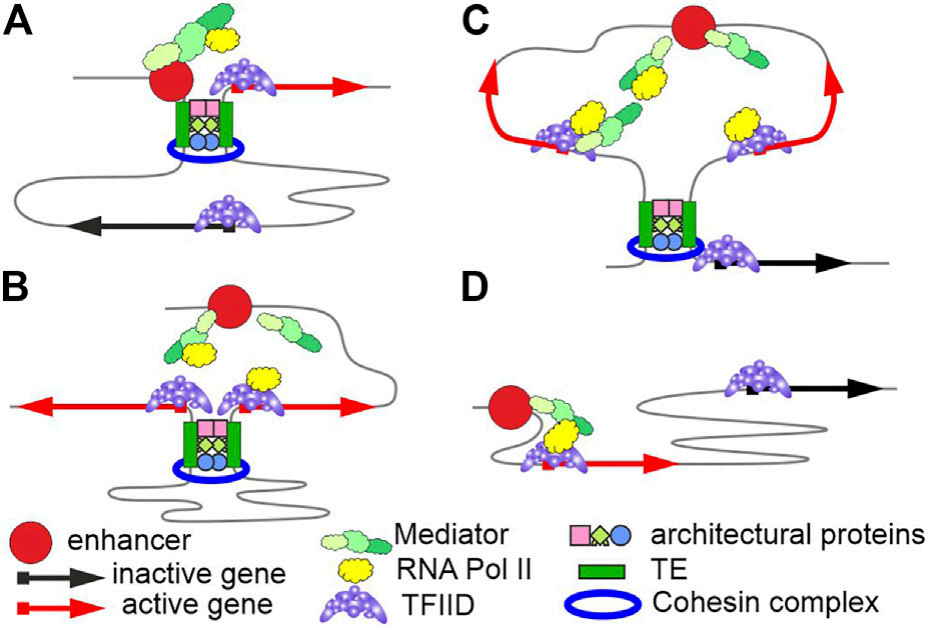
Variants of functional interactions between enhancers and promoters. The model suggests that promoters and enhancers do not independently maintain their specific long-range interactions. A special class of tethering elements (TEs) may maintain the long-range interactions. Cohesin complexes sustain the interactions between TEs in mammals. **(A)** TEs occur in an enhancer and a promoter and ensure a specific long-range interaction between them. **(B)** TEs occur in promoters, bringing them close together and co-activation by a single enhancer. **(C)** Interactions between TEs bring groups of enhancers and promoters close together, and the elements are simultaneously activated with different efficiencies, which depend on the promoter strength and the distance to an enhancer. **(D)** The nearest promoters are activated by enhancers in the absence of TEs.

Many modern studies have shown that at least some functionally interacting enhancers and promoters do not form stable contacts (Hafner and Boettiger, 2022). To explain, it is assumed that promoters must be located in a certain zone of action of enhancers. During activation, enhancers usually recruit several complexes possessing acetyltransferase (p300/CBP) and methyltransferase (Mll3/Mll4/COMPASS) activities (Cenik and Shilatifard, 2021) and the Mediator complex (Richter et al., 2022), which facilitates assembly of the preinitiation complex and is involved in recruiting RNA polymerase II to the promoter. Mediator complexes are presumably concentrated on enhancers and, upon binding with RNA polymerase II, move to promoters and bind with the general TFIID complex. RNA polymerase II recruitment to enhancers by Mediator agrees with transcription that is initiated on enhancers and yields eRNAs (Sartorelli and Lauberth, 2020). A higher concentration of Mediator complexes with RNA polymerase II thus occurs around enhancers, stimulating transcription from neighbor promoters. Once transcription is initiated on a promoter, Mediator is possibly released from its complex with TFIID and moves to an enhancer. An alternative model (Karr et al., 2022) suggests that the p300/CBP complex bound to an enhancer acetylates and thus activates TFs. A higher concentration of active TFs around the enhancer increases the activities of neighbor promoters. Several variants of transcription activation are possible to assume depending on the relative arrangement of TEs, enhancers, and promoters (Figure 1).

## 3 The role of cohesin in chromosome organization in mammals and *Drosophila*

The cohesin complex is highly conserved in eukaryotes. Its main function is to hold sister chromatids together during mitosis and meiosis (Dorsett, 2019; Davidson and Peters, 2021). There are four core subunits in the complex: ATPases SMC1 and SMC3, RAD21, and STAG1 or STAG2. SMC1 and SMC3 form a heterodimeric ring, interacting simultaneously through their N- and C- termini, with the hinge domain on one side and the ATPase domain on the other. RAD21 interacts with the terminal domains of SMC1 and SMC3 to hold them together in the absence of ATP. STAGs interact with RAD21 and SMCs.

**FIGURE 2:**
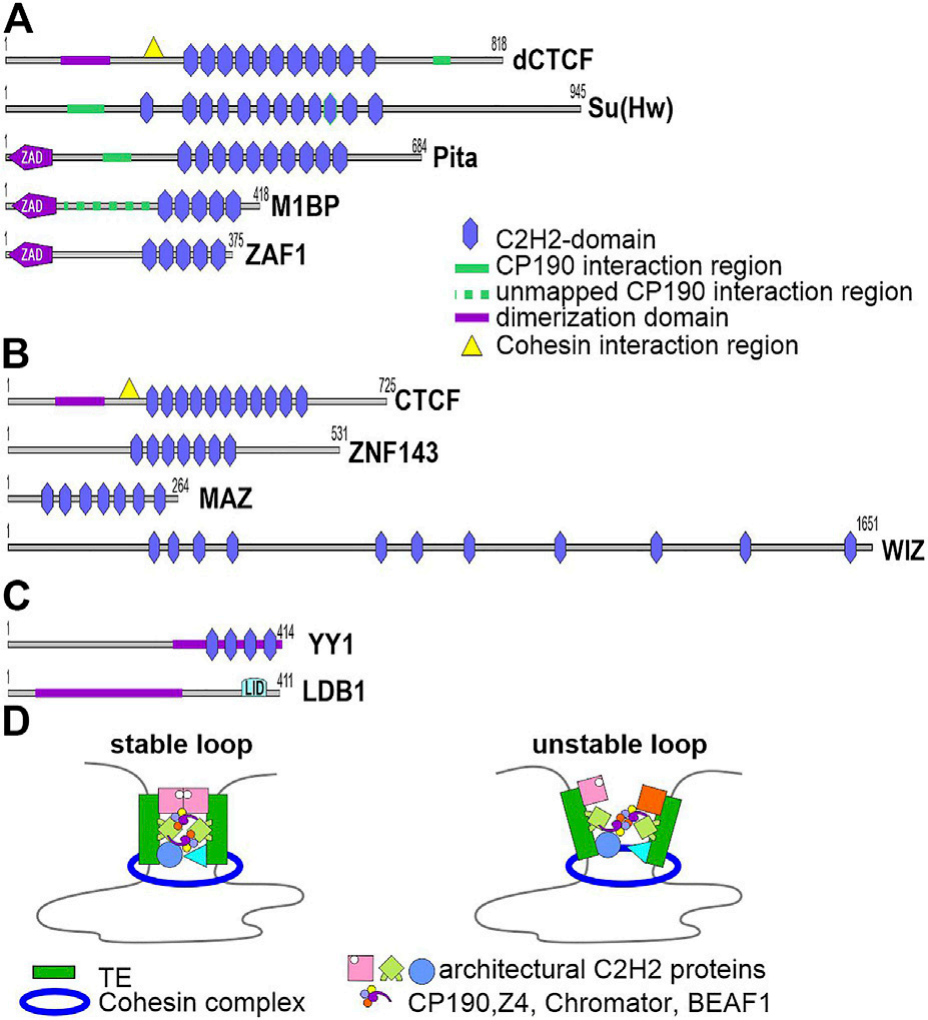
Main *Drosophila* C2H2 architectural proteins and mammalian proteins involved in long-range interactions. **(A)** Schematic structures of the *Drosophila* C2H2 architectural proteins studied in most detail: dСTCF, Su(Hw), Pita, M1BP, and ZAF1. Unstructured regions interacting with CP190 were precisely mapped in CTCF, Su(Hw) and Pita, but not in M1BP. **(B)** Schematic structures of human CTCF and the C2H2 proteins ZNF143, MAZ, and WIZ, which were shown to cooperate with CTCF in organizing the chromosome architecture. A motif responsible for interactions with the cohesin complex was mapped only in CTCF. **(C)** LDB1 and YY1, which maintain specific enhancer–promoter interactions in mammals. Homodimerization domains are indicated. **(D)** Currently, there are no experimental data showing that cohesin-mediated loop extrusion and protein-protein interactions between transcription factors cooperatively form chromatin loops between regulatory elements. However, a model can be proposed according to which architectural C2H2 proteins with associated transcription factors form regulatory elements that limit cohesin-mediated loop extrusion through multiple interactions with different cohesive subunits. Homodimerization between C2H2 architectural proteins and interaction between associated transcription factors stabilize the chromatin loop.

In mammals, the cohesin complex, together with CTCF, is involved in organizing long-range enhancer–promoter interactions and forming the TAD boundaries in interphase nuclei (Sikorska and Sexton, 2020; Jerkovic and Cavalli, 2021). A characteristic feature in the structure of the CTCF protein is the presence in the central part of a cluster consisting of 11 domains of C2H2 type zinc fingers (Figure 2). Five C2H2 domains of CTCF specifically bind to a 15-bp motif, which is conserved among most animals (Hashimoto et al., 2017). A conserved YxF motif, which interacts with the RAD21/STAG cohesin subcomplex, was found at the N terminus of human CTCF (Li et al., 2020).

A common model suggests that the cohesin complex, after binding to chromatin, begins DNA extrusion with the formation of a chromatin loop. CTCF-binding sites block its progress and fix the chromatin domain boundaries (Fudenberg et al., 2016). When CTCF is inactivated, cohesin complexes are relocated from CTCF-binding sites to active gene promoters and the TAD configuration is partly destroyed (Nora et al., 2017; Rao et al., 2017). CTCF mutations and deletions that affect the interaction with the cohesin complex also dramatically distort both long-range interactions and TADs (Li et al., 2020; Pugacheva et al., 2020).

Studies in *Drosophila* initially revealed the role of the cohesin complex in regulating long-range enhancer–promoter interactions (Rollins et al., 1999; Dorsett et al., 2005). The cohesin complex is detectable in regions of regulatory elements, primarily enhancers and promoters of active genes, in open chromatin (Dorsett, 2019; Pherson et al., 2019). However, in *Drosophila* remains unstudied how the inactivation of the cohesin complex affects the formation of TAD and long-range interactions between regulatory elements. *Drosophila* CTCF (dCTCF) lost some of its functions, and *ctcf* null mutants are consequently viable (Kyrchanova et al., 2021). dCTCF has fewer than 1,000 binding sites that co-localize with only a minor part of TAD boundaries (Fudenberg and Nora, 2021; Kaushal et al., 2021). The role that the cohesin complex plays in *Drosophila* chromosome architecture needs further investigation.

## 4 The role of cooperation between architectural proteins in the organization of functional regulatory elements

Several architectural proteins similar to dCTCF in having C2H2 domain clusters are encoded in the *Drosophila* genome. Usually 4-5 C2H2 domains provide specific binding of proteins with long 12–15 bp motifs (Maksimenko et al., 2021). Apart from dCTCF, Pita, Zw5, Zipic, M1BP, and Su(Hw) were best studied in this class of architectural C2H2 proteins (Figure 2A). Their binding sites are located predominantly in gene promoters (Schwartz et al., 2012; Li and Gilmour, 2013; Maksimenko et al., 2015; Zolotarev et al., 2016; Baxley et al., 2017).

The functional role of architectural proteins in insulation is well illustrated by the regulatory region of the *Bithorax* complex, which includes three homeotic genes (Kyrchanova et al., 2015; Maeda and Karch, 2015). Insulators occur at the boundaries of autonomous regulatory domains, which each determine expression of one of the three *Hox* genes in only one of the *Drosophila* segments. Each insulator has one or two motifs for Su(Hw), Pita, and CTCF. For example, the *Mcp* insulator harbors the binding sites for Pita and dCTCF, and the *Fub* insulator has one binding site for dCTCF, one for Pita, and two for Su(Hw) (Kyrchanova et al., 2017, 2020). *In vivo* editing of the *Bithorax* complex showed that at least four binding sites for the same or different architectural proteins make an active insulator. The efficiency of dCTCF binding to a respective site depends on whether a Pita-binding site occurs in its vicinity. Thus, the C2H2 architectural proteins bind to chromatin and work cooperatively and can functionally substitute each other in forming the boundaries of the autonomous regulatory domains in the *Bithorax* locus. A similar collaboration of many closely spaced CTCF sites in organizing of robust boundaries was recently demonstrated in transgenic mouse assay (Anania et al., 2022).

The *Drosophila* C2H2 architectural proteins also work cooperatively to form active promoters. A large group of architectural C2H2 proteins, including M1BP, CTCF, Su(Hw), and Pita, ensures the promoter localization of the TF CP190 (Bag et al., 2021; Kyrchanova et al., 2021; Sabirov et al., 2021). CP190 is important for recruiting the transcription complexes to promoters (Maksimenko et al., 2020). Transgenic lines were constructed to express dCTCF and Pita mutants incapable of interacting with CP190. CP190 binding to CTCF- and Pita-dependent promoters was not distorted, indicating that other, still unknown C2H2 architectural protein recruited CP190 (Kyrchanova et al., 2021; Sabirov et al., 2021).

Experimental data are accumulating to demonstrate that other DNA-binding TFs may act independently or cooperate with CTCF to maintain long-range interactions in mammals (Hsieh et al., 2020; Krietenstein et al., 2020; Liu et al., 2021; Taylor et al., 2022). ZNF143, MAZ, and WIZ were identified as C2H2 partners of CTCF and, like CTCF, are necessary for mammalian embryo development (Figure 2B).

ZNF143 has seven C2H2 domains, which determine its specific binding to 15-bp motifs (Bailey et al., 2015). ZNF143 is involved in opening chromatin and recruiting transcription-activating complexes to gene promoters (Sathyan et al., 2019). Closely spaced motifs for CTCF and ZNF143 were found in a large group of mouse enhancers and promoters (Zhou et al., 2021). CTCF binding depends on the presence of a ZNF143 site, and the two proteins cooperatively contribute to the formation of long-range enhancer–promoter interactions (Bailey et al., 2015; Zhou et al., 2021).

MAZ helps CTCF to organize the functional boundaries of the *Hox* genes (Ortabozkoyun et al., 2022). MAZ predominantly binds to gene promoters through cluster of the C2H2 domains. Approximately 20% of MAZ-binding sites co-localize with CTCF sites in mouse ESCs (Ortabozkoyun et al., 2022). MAZ facilitates CTCF binding to chromatin in certain genome regions (Xiao et al., 2021), and the two proteins cooperatively regulate transcription of several genes (Ortabozkoyun et al., 2022). MAZ directly interacts with Rad21 through an unidentified domain (Xiao et al., 2021).

WIZ interacts with CTCF and an unidentified subunit of the cohesin complex (Justice et al., 2020). The cohesin complex can be fixed at specific chromatin sites through cooperation between CTCF, WIZ, and MAZ, which likely interact with different regions of the cohesin subunits. Inactivation of MAZ, WIZ, or ZNF143 only slightly distorts certain enhancer–promoter contacts within TADs (Justice et al., 2020; Xiao et al., 2021; Ortabozkoyun et al., 2022). Thus, the architectural proteins act cooperatively to form the chromosome architecture and to organize the functional regulatory elements, such as promoters or insulators, in both mammals and *Drosophila*.

## 5 Role of homodimerization domains in maintaining distant interactions between enhancers and promoters

Studies with transgenic *Drosophila* lines showed that pairing of identical insulators can maintain super-long-distance interactions between enhancers/silencers and reporter gene promoters (Fujioka et al., 2016; Kyrchanova and Georgiev, 2021). A model advanced to explain the stabilization of super-long-distance interactions suggests that insulators consist of unique combinations of binding sites for several architectural C2H2 proteins capable of homodimerization (Kyrchanova and Georgiev, 2014, 2021). The interaction efficiency of two identical insulators is therefore far higher than that of heterologous insulators, which harbor different combinations of motifs for architectural C2H2 proteins. A zinc finger-associated domain (ZAD), which forms homodimers, was found indeed at the N ends of the majority of known *Drosophila* architectural C2H2 proteins. In *Drosophila melanogaster,* 98 C2H2 proteins with N-terminal ZAD are capable of preferential homodimerization (Bonchuk et al., 2021, 2022). The ZAD proteins Pita, Zipic, and Zw5 maintained specific long-range interactions in model transgenic lines (Zolotarev et al., 2016). Most of the ZAD C2H2 proteins probably perform architectural functions because a small protein chosen arbitrarily and termed ZAF1 (Figure 2A) efficiently performs the insulator functions and maintains distance interactions in transgenic systems (Maksimenko et al., 2020).

There are a number of experimental data that CP190, in cooperation with BEAF, Chromator and Z4, can participate in the organization of TADs (Ramírez et al., 2018; Wang et al., 2018; Pal et al., 2019). These proteins interact with each other, can homodimerize, and bind to large part of hk promoters (Gan et al., 2011; Vogelmann et al., 2014; Cubeñas-Potts et al., 2017; Dong et al., 2020; Melnikova et al., 2021). The main function of the proteins is the recruitment of transcription complexes and it is assumed that they can simultaneously participate in maintaining long-distance interactions between hk promoters.

An unstructured domain capable of forming tetramers was mapped at the N end of *Drosophila* CTCF (Bonchuk et al., 2015, 2020). The domain is critical for functional activity of CTCF *in vivo* (Bonchuk et al., 2015). Interestingly, almost all known CTCFs of various animals, including human, have an unstructured domain capable of homodimerization at their N ends (Bonchuk et al., 2020). It is still unclear what is the role of the dimerization domains in mammalian CTCF proteins.

In mammals, the LIM dimerization domain (Figure 2B) was well studied in domain-binding factor 1 (LBD1), which possibly organizes the long-range enhancer–promoter interactions (Krivega and Dean, 2016). LDB1 binds to chromatin through LIM-family DNA-binding TFs, and its N-terminal domain forms a stable homodimer (Wang et al., 2020). LIM-family TFs bind predominantly to enhancers and promoters and, according to a model, recruit LDB1 to them, and the N-terminal domain of LDB1 maintains long-distance enhancer–promoter interactions (Deng et al., 2012; Krivega et al., 2014). The *Drosophila* LDB1 homolog Chip contributes to organizing long-range interactions between the enhancer and promoter of *cut* (Morcillo et al., 1997).

The small (414 a. a.) conserved protein Ying Yang 1 (YY1, Figure 2B) has four zinc fingers and binds predominantly to enhancers and promoters, consistently with its potential role in long-distance interactions (Deng et al., 2012; Weintraub et al., 2017). YY1 inactivation appreciably decreased the interactions between certain enhancers and promoters in cell lines. Hydrophobic region 200–226 is responsible for YY1 multimerization and acts in cooperation with the C2H2 domains (López-Perrote et al., 2014; Qiao et al., 2022). The C2H2 cluster determines the YY1 binding to a specific motif in chromatin (López-Perrote et al., 2014). A potential role of the C2H2 domains of YY1 in organizing long-range interactions is of principal importance because only a minor part of more than 700 C2H2 proteins have proven N-terminal dimerization domains (Maksimenko et al., 2021). Interestingly, the Su(Hw) classical architectural protein with 12 C2H2 domains lacks N-terminal dimerization domains, but maintains long-distance interactions in model systems. Its C2H2 domains that are not involved in DNA binding may ensure the interaction between Su(Hw) molecules located at different ends of a chromatin loop, as is assumed for mammalian YY1 (Weintraub et al., 2017).

## 6 Conclusion

The views of chromosome architecture and long-distance enhancer–promoter interactions in higher eukaryotes substantially changed with recent development of more precise techniques (Jerkovic and Cavalli, 2021; Hafner and Boettiger, 2022). It becomes clear that chromatin looping cannot be a main mechanism producing independent transcription domains, as was demonstrated with model systems in transgenic *Drosophila* lines (Savitskaya et al., 2006; Maksimenko et al., 2008; Kyrchanova et al., 2013). To functionally interact, enhancers and promoters can occur a certain distance apart, rather than in a tight association assumed until recently. Data are accumulating that other C2H2 architectural proteins act along with CTCF to organize long-distance interactions in mammals and *Drosophila*. It is very likely that there are many mammalian C2H2 architectural proteins, and most of them have not yet been studied. It can be assumed that architectural C2H2 proteins in cooperation with associated chromatin proteins can initially form chromatin loops as a result of the restriction of DNA extrusion by cohesin and stabilize the newly created chromatin loops using protein-protein interactions between homodimerizing domains (Figure 2B). New study (Dequeker et al., 2022) has shown that mini-chromosome maintenance (MCM) complex can also function as barriers that restrict cohesin-mediated loop extrusion during G1 phase. Recently, more and more breakthrough results have appeared that will soon contribute to understanding how the architecture of interphase chromosomes is formed.
